# Serum Levels of Anticyclic Citrullinated Peptide Antibodies, Interleukin-6, Tumor Necrosis Factor-***α***, and C-Reactive Protein Are Associated with Increased Carotid Intima-Media Thickness: A Cross-Sectional Analysis of a Cohort of Rheumatoid Arthritis Patients without Cardiovascular Risk Factors

**DOI:** 10.1155/2015/342649

**Published:** 2015-03-02

**Authors:** Mónica Vázquez-Del Mercado, Lourdes Nuñez-Atahualpa, Mauricio Figueroa-Sánchez, Eduardo Gómez-Bañuelos, Alberto Daniel Rocha-Muñoz, Beatriz Teresita Martín-Márquez, Esther Guadalupe Corona-Sanchez, Erika Aurora Martínez-García, Héctor Macias-Reyes, Laura Gonzalez-Lopez, Jorge Ivan Gamez-Nava, Rosa Elena Navarro-Hernandez, María Alejandra Nuñez-Atahualpa, Javier Andrade-Garduño

**Affiliations:** ^1^Instituto de Investigación en Reumatología y del Sistema Músculo Esquelético, Centro Universitario de Ciencias de la Salud, Universidad de Guadalajara, Sierra Mojada No. 950, Colonia Independencia, 44340 Guadalajara, JAL, Mexico; ^2^Departamento de Reumatología, Hospital Civil “Dr. Juan I. Menchaca”, Universidad de Guadalajara, Salvador de Quevedo No. 750, 44100 Guadalajara, JAL, Mexico; ^3^Hospital Civil de Guadalajara “Fray Antonio Alcalde”, Universidad de Guadalajara, Hospital No. 278, 44280 Guadalajara, JAL, Mexico; ^4^Departamento de Fisiología, Centro Universitario de Ciencias de la Salud, Universidad de Guadalajara, Sierra Mojada No. 950, Colonia Independencia, 44340 Guadalajara, JAL, Mexico; ^5^Departamento de Medicina Interna-Reumatología, Hospital General Regional No. 110, Instituto Mexicano del Seguro Social, Circunvalación Oblatos No. 2212, Colonia Oblatos, 44700 Guadalajara, JAL, Mexico; ^6^Unidad de Investigacion, Unidad Medica de Alta Especialidad, Hospital de Especialidades del Centro Médico Nacional de Occidente, Instituto Mexicano del Seguro Social, Belisario Domínguez No. 1000, Independencia Oriente, 44340 Guadalajara, JAL, Mexico; ^7^Facultad de Medicina, Universidad Autónoma de Guadalajara, Avenida Patria 1201, Lomas del Valle, 45129 Zapopan, JAL, Mexico

## Abstract

The main cause of death in rheumatoid arthritis (RA) is cardiovascular events. We evaluated the relationship of anticyclic citrullinated peptide (anti-CCP) antibody levels with increased carotid intima-media thickness (cIMT) in RA patients. *Methods*. Forty-five anti-CCP positive and 37 anti-CCP negative RA patients, and 62 healthy controls (HC) were studied. All groups were assessed for atherogenic index of plasma (AIP) and cIMT. Anti-CCP, C-reactive protein (CRP), and levels of tumor necrosis factor alpha (TNF*α*) and interleukin-6 (IL-6) were measured by enzyme-linked immunosorbent assay (ELISA). *Results*. The anti-CCP positive RA patients showed increased cIMT compared to HC and anti-CCP negative (*P* < 0.001). Anti-CCP positive versus anti-CCP negative RA patients, had increased AIP, TNF*α* and IL-6 (*P* < 0.01), and lower levels of high density lipoprotein cholesterol (HDL-c) (*P* = 0.02). The cIMT correlated with levels of anti-CCP (*r* = 0.513, *P* = 0.001), CRP (*r* = 0.799, *P* < 0.001), TNF*α* (*r* = 0.642, *P* = 0.001), and IL-6 (*r* = 0.751, *P* < 0.001). In multiple regression analysis, cIMT was associated with CRP (*P* < 0.001) and anti-CCP levels (*P* = 0.03). *Conclusions*. Levels of anti-CCP and CRP are associated with increased cIMT and cardiovascular risk supporting a clinical role of the measurement of cIMT in RA in predicting and preventing cardiovascular events.

## 1. Introduction

Rheumatoid arthritis (RA) is a chronic autoimmune disease with a major component of inflammatory process [1, 2]. The main cause of death in these patients is cardiovascular events, which lead to a decreased life expectancy by 3 to 10 years [3, 4].

Cytokines play an important role in the regulation of inflammatory process and severity and progression of RA. Macrophages and lymphocytes are considered the main mediators of inflammation as these are the main source of interleukin-6 (IL-6) and tumor necrosis factor alpha (TNF*α*), the two major cytokines implicated in the pathogenesis of RA [5]. IL-6 may increase the risk of atherosclerosis mediated by endothelial damage, as well as increased carotid intima-media thickness (cIMT) [6].

Anticyclic citrullinated peptide (anti-CCP) antibodies are associated with the pathogenesis, clinical expression, and cardiovascular risk in RA [7–9]. Sokolove et al. [10] demonstrated by immunohistochemistry the presence of citrullinated proteins within the damaged endothelium in atherosclerotic plaque. RA patients positive for anti-CCP or rheumatoid factor (RF) have more endothelial dysfunction [9]. So far, it has been shown that positivity of anti-CCP antibodies is associated with the increased cIMT [11–15].

The measurement of cIMT >0.6 mm is a marker of atherosclerosis and has been suggested as a surrogate marker of subclinical atherosclerotic disease [2]. The ultrasound findings of carotid tissue are considered by some authors to be a mirror of the coronary arteries' condition [16]. The cIMT is strongly correlated with cardiovascular disease (CVD) risk factors [1, 16, 17] and clinical coronary events [1, 2, 18, 19]. RA patients with values of 0.9 mm in cIMT or the presence of atherosclerotic plaques is highly related with high CVD risk [2, 20, 21].

The cIMT in RA patients has been associated with increased levels of inflammatory molecules in independent studies focused on particular markers [2, 16, 32]. Scarce studies have explored the presence of cytokine expression and anti-CCP antibodies. The aim of this work was to evaluate the relationship of levels of anti-CCP antibodies, inflammation markers, and subclinical atherosclerosis measured by cIMT in RA patients without comorbidities.

## 2. Methods

### 2.1. Study Design

It is a cross-sectional study.

#### 2.1.1. Patients

The study population was recruited over a period of 2 years from 2010 to 2012 and included 82 patients with RA attending an outpatient rheumatology service of the Hospital Civil “Dr. Juan I. Menchaca” of the Universidad de Guadalajara, Jalisco. To be eligible for the study, patients had to be 18 years or older and to have met the American College of Rheumatology criteria (1987) and ACR/European League against Rheumatism (EULAR) 2010 [33, 34]. For the healthy control group (HC) we included blood donors without rheumatic disease matched by gender and age and were assessed by cardiovascular risk profile and family history of CVD. The exclusion criteria for both groups were previous or current smoking history, ischemic CVD, hypertension, diabetes mellitus, thyroid disease, renal impairment, malignancy, hepatic disease, and hyperlipidemia. We also excluded patients previously treated with high doses of steroids (>10 mg/day prednisone or equivalent, including intravenous administration).

#### 2.1.2. Definition and Assessment of cIMT

The cIMT was assessed according to the recommendations defined by the Mannheim Carotid Intima-Media Thickness and Plaque Consensus (2004–2006–2011) [17]. The cIMT was measured using a high-resolution B-mode ultrasound (US) (PHILIPS, Saronno, Italy) with a 9 MHz transducer. Briefly, with the subject in the supine position in a semidark room, longitudinal scanning was performed from the common carotid artery (CCA) to the cranial entry of the internal carotid artery (ICA). The evaluated segments of the left and right carotid arteries were the CCA, carotid bifurcation (BF), and ICA. Two segments of the CCA, one from the BF, and two from the ICA were measured, providing a total of 10 measurements per individual. Mean cIMT values were calculated for each segment of the carotid arteries. Hence, five cIMT values were obtained. All measurements were performed by a single operator.

The ultrasound images and parameters were evaluated by two expert radiologists (LNA, MFS) blinded to the clinical characteristics of patients. The cIMT was determined as a double-line pattern visualized by echography on both walls of the CCA in a longitudinal image. Two parallel lines, which consist of the leading edges of two anatomical boundaries, form the lumen-intima and media-adventitia interfaces as demonstrated in a previous study [17].

#### 2.1.3. Clinical Assessment

A structured questionnaire was applied to each patient in order to evaluate demographic and clinical variables including disease duration and treatment. The clinical evaluation was performed by trained personnel; RA disease activity was measured by disease activity score (DAS) 28 [35], erythrocyte sedimentation rate (ESR), and posteroanterior radiographs of the hands obtained at the time of recruitment. The degree of RA progression was assessed by the Steinbrocker score of the metacarpophalangeal (MCP) joints [36].

#### 2.1.4. Anti-CCP and Other Laboratory Measurements

ESR was measured using the Wintrobe method. The CRP levels were calculated by nephelometry; total cholesterol (TC), triglycerides (Tg), high density lipoprotein cholesterol (HDL-c), and low density lipoprotein cholesterol (LDL-c) were measured by standard techniques after centrifugation of blood samples. Cardiovascular risk ratio was calculated using atherogenic index of plasma (AIP) which was defined as TC/HDL-c [37].

Venous blood samples were collected immediately at the moment of the clinical assessment. Serum was obtained by centrifugation of whole blood at 2,000 rpm for 15 minutes, and aliquots with serum were stored at −70°C for no longer than 6 months and were used for the determination of anti-CCP antibodies, IL-6, and TNF*α* by enzyme-linked immunosorbent assay (ELISA) (R&D Systems, Bender MedSystem).

#### 2.1.5. Statistical Analysis

Variables were tested for normality using the Kolmogorov-Smirnov test. Normally distributed values are presented as means with standard deviations (SD), or percentages as appropriate. Between-group differences were estimated by independent-sample Student's *t*-test and ANOVA test. Chi-square test (or Fisher's exact test) was used for comparing categorical variables. Pearson's correlation coefficient was calculated for cIMT, DAS28, CRP, levels of anti-CCP, IL-6, and TNF*α*. Risk of abnormal cIMT (>0.6 mm) in patients was quantified by an odds ratio (OR) with a 95% confidence interval. Multiple linear regression analysis was performed to assess independent associations between cIMT, clinical evaluation, and laboratory measurement. All data were analyzed using SPSS 16.0 software (SPSS Inc, Chicago, IL), considering a two-tailed level of *P* < 0.05 to be statistically significant for univariate and multivariate analysis.

#### 2.1.6. Ethical Approval

This protocol was approved by the IRB Committee with the register 1068/10 of the Hospital Civil “Dr. Juan I. Menchaca” of the Universidad de Guadalajara, following Helsinki declaration.

## 3. Results

Since our main objective was to detect an increased cIMT suggesting subclinical atherosclerosis in RA subjects, the cIMT was assessed by high-resolution B-mode US in 62 HC, 45 RA patients anti-CCP positive, and 37 RA patients anti-CCP negative.

### 3.1. RA Patients Had Increased cIMT

**Figure 1:**
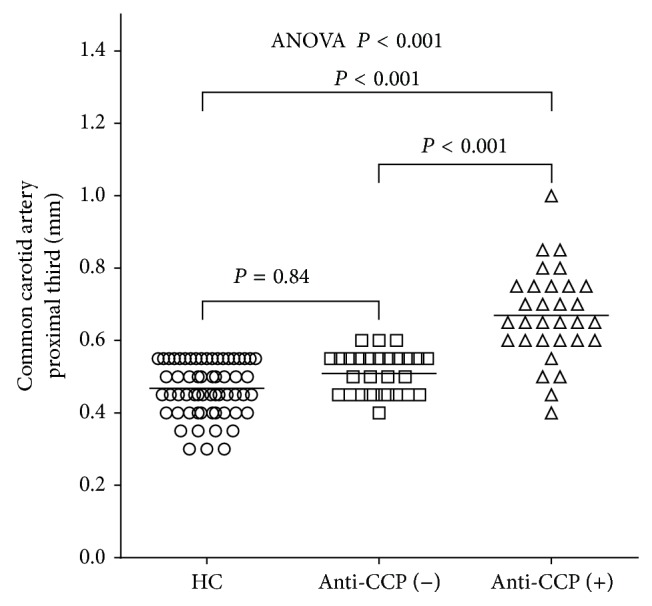
Carotid intima-media thickness (cIMT) in patients with rheumatoid arthritis (RA) classified by anti-CCP antibodies, compared with healthy controls (HC). Horizontal bars indicate the median. ANOVA *P* values indicate the significance of the overall trend while comparisons between groups are compared by Scheffé's post hoc test.

The US assessment of the carotid artery between HC and patients with RA is shown in Table 1. Remarkably, the increased thickness of cIMT and carotid segments was significant between HC and RA (*P* ≤ 0.01), but not in the presence of carotid plaques (*P* = 0.83). The segments measured by US were thicker in CCA, bulb, and ICA in RA patients. The mean value of cIMT was higher among the RA anti-CCP positive patients when compared with the anti-CCP negative group and HC, *P* < 0.001 (Figure 1). The OR of an increased cIMT (>0.6 mm) in RA patients was 5.68 (95% CI 2.12–15.24, *P* < 0.001) compared to HC. An OR of 4.83 (95% CI 2.27–9.81, *P* < 0.001) was obtained when comparing RA anti-CCP positive versus RA anti-CCP negative.

### 3.2. Increased Cardiovascular Risk Is Associated with Clinical and Laboratory Characteristics in RA Patients

The clinical and laboratory findings in the RA study groups are summarized in Table 2. In comparison between anti-CCP negative (*n* = 37) versus anti-CCP positive (*n* = 45) patients, the anti-CCP positive had higher DAS28 (1.43 ± 0.95 versus 3.14 ± 0.44 units, *P* = 0.05) with a Steinbrocker radiological stage III or IV (0 versus 15.6%, *P* = 0.01).

Higher concentrations of serum lipids were found in anti-CCP positive compared to anti-CCP negative patients. However, we could not find differences between RA and HC in lipid profile (data not shown). Anti-CCP positive patients had a moderate cardiovascular risk according to the AIP. Levels of RF, ESR, CRP, TNF*α*, and IL-6 were increased in the RA anti-CCP positive group. When treatment was evaluated in these RA patients, the use of methotrexate was more frequent in anti-CCP positive patients (*P* = 0.04).

### 3.3. Correlations between cIMT and Clinical and Laboratory Characteristics in RA Patients

There was a correlation coefficient (*r*) ≥ 0.3 between age, DAS28, and Tg. In contrast, the correlation coefficients between the cIMT and AIP, TC, CRP, TNF*α*, and IL-6 were ≥0.600 (Table 3).

### 3.4. Multivariate Analysis

To determine whether demographic, clinical, and serological variables were potential confounders or effect modifiers, we carried out a univariate linear regression analysis to determine which were most significantly associated with cIMT. Variables with a* P* value of 0.2 or less were chosen for inclusion in further multivariate analyses. The results of multivariate linear regression analysis of clinical variables associated and the measurement of cIMT are shown in Table 4. After adjustment for age and disease duration, the variables associated with an increase in cIMT were CRP (*P* = 0.05) and anti-CCP (*P* = 0.005) (Model 1); after inclusion of RF and DAS28 score in Model 2, only CRP (*P* = 0.05) and anti-CCP (*P* = 0.009) were positively associated with cIMT. No significant relationships were identified with other clinical variables. When we considered TNF*α* and IL-6 levels in Model 3, the variables that remained associated with cIMT were CRP (*P* < 0.001) and anti-CCP (*P* = 0.03). If we excluded the anti-CCP levels from the model, this variable by itself is responsible for no clinical association with the rest of parameters included, which may be interpreted as a cardiovascular risk factor to be evaluated along with cIMT.

## 4. Discussion

The cardiovascular risk in RA increases with cIMT, suggesting that the pathophysiological mechanisms that underlie the progression of carotid injury in RA may differ from the general population. In order to exclude the influence of other comorbidities in the development of cardiovascular risk in RA patients, we decided to apply strict exclusion criteria for the present study, excluding obesity, smoking, hypertension, diabetes, and other comorbidities like thyroid, liver, and renal disease. Thus, we were able to discriminate the influence of other independent risk factors previously reported for increased cIMT, such as age > 65 years old (OR 3.7), male gender (OR 1.9), smokers (OR 2.2), hypertension (OR 5.0), and diabetes (OR 2.4) [12, 38, 39]. In our study, RA was an independent cardiovascular risk factor associated with a 4-fold risk for increased cIMT, in conjunction with anti-CCP antibody levels with an OR of 4.8 (95% CI 2.27–9.85).

Although the biological role of anti-CCP is controversial, citrullinated proteins have been identified in various tissues affected in RA, such as lung tissue, vascular endothelium, endocardium, and oral mucosa [10, 40, 41]. López-Longo et al. [42] found that anti-CCP antibodies are associated with increased risk of ischemic heart disease. Also an association of anti-CCP antibodies with endothelial dysfunction has been described in RA patients [42, 43].

Despite several reports about anti-CCP and cIMT in RA (Table 5), only in few the association between anti-CCP antibodies and subclinical atherosclerosis has been evaluated. Notwithstanding, this association has only been evaluated from a “qualitative” point of view and not taking into account the effect of the serum levels of anti-CCP antibodies. In this study we demonstrated by multiple linear regression analysis, an independent association between serum levels of anti-CCP antibodies and cIMT after adjustment for age, gender, and disease activity. According to our results, for every unit of anti-CCP antibodies, there would be an increment of 0.001 mm in the cIMT (*β* coefficient for anti-CCP in multiple regression analysis, Table 4). These findings suggest a possible role of anti-CCP antibodies in the pathogenesis of atherosclerosis in RA, but also the relevance of their role in the prediction of cardiovascular risk in this group of patients.

In the present study, we reported that RA patients with high levels of anti-CCP antibodies have a poor clinical prognosis and subclinical cardiovascular risk, based on increased cIMT, CRP, and high levels of proinflammatory molecules such as TNF*α* and IL-6. Serum TNF*α* and IL-6 were strongly correlated with cIMT (Table 3) [44]. Furthermore, the anti-CCP positive patients had a more atherogenic lipid profile characterized by lower HDL-c and a high AIP (Table 2).

It is well known that cIMT reflects the integrity of coronary arteries [16, 45]. In this scenario we might suggest that our RA patients have subclinical atherosclerosis given that we found a greater proportion of affected carotid segments, 3 : 1, when compared with HC [43].

It is important to highlight that, unlike the present study, carotid plaques have been more commonly observed in RA patients versus matched HC. Gonzalez-Juanatey et al. [46] reported a higher frequency of carotid plaques in RA without comorbidities (34%, *n* = 16) versus HC (15%, *n* = 7), *P* = 0.031. RA patients with carotid plaques were older than HC and had longer disease duration, higher cIMT, and more frequently extra-articular manifestations.

An important issue related to the development of atherosclerosis in RA patients is to look for the causes of endothelial dysfunction. In this context, proinflammatory cytokines such as IL-6 and TNF*α* have been correlated with cIMT [47].

On the other hand, one point that should be addressed as possible contributor to accelerated atherosclerosis is the genetic background. In this context, HLA-DRB1^*^0404 is related with increased CVD risk and high levels of anti-CCP antibodies [48–50].

Genes for proinflammatory cytokines such as IL-6 and TNF*α* are recognized as inducers of systemic and local manifestations of RA. The contribution of IL-6-174 GG genotype in the development of severe endothelial dysfunction by flow-mediated endothelium-dependent vasodilatation in patients with RA was reported by Palomino-Morales et al. [51].

In addition, the allele A of TNF*α* polymorphism −308G>A (rs1800629) has been associated with a higher risk of CVD in RA patients who are carriers of at least one copy of the shared epitope [52]. These results highlight the potential implication of TNF*α* in the mechanisms associated with CVD in RA, as well as the improvement in CVD risk in patients treated with TNF*α* blockers. Gonzalez-Juanatey et al. [53] showed that short term therapy with adalimumab improved the endothelial dependent vasodilatation as well as acute phase reactants, disease activity, and AIP. On the other hand, Gonzalez-Gay et al. [54] showed that infliximab infusion was able to decrease proinflammatory cytokines such as resistin in RA patients, measured before and after (120 minutes) infliximab infusion. Also, they showed the reduction in acute phase reactants such as ESR, platelet count, and CRP when compared from disease diagnosis to time of the study.

We found an association between CRP levels and cIMT. This observation is in accordance with a previous report by Gonzalez-Gay et al. [55] in a retrospective study of RA patients without comorbidities, treated with at least one of disease-modifying antirheumatic drugs (DMARDs) during 5 years. They found a positive correlation between the maximum observed CRP (during follow-up) and current cIMT (*r* = 0.37, *P* < 0.009). This phenomenon was also observed in patients with CRP > 10 mg/L (*r* = 0.316, *P* = 0.031).

Finally, in our multivariate analysis, higher levels of CRP and levels of anti-CCP antibodies remained associated with the cIMT, independent of age and disease duration, suggesting that the possible damage to vascular endothelium in the carotid arteries is a subclinical but active process in RA. In our study, anti-CCP antibodies explained up to 80% of the variability in the cIMT; this observation has not been previously shown in studies regarding atherosclerosis and RA; we consider this observation as a strength of our study (Table 4).

One caveat of our study is the cross-sectional design, so we cannot establish a biological explanation enough to sustain the link of anti-CCP antibodies and endothelial damage; so far the anti-CCP antibodies are only considered as a clinical marker of disease. We cannot rule out the influence of cumulative disease activity measured by DAS28 and CRP in the development of subclinical atherosclerosis given the cross-sectional nature of our study.

Further studies are needed to evaluate the possible relationship between serum levels of anti-CCP and future cardiovascular events and assess whether these markers are predictive of a worse CVD outcome. A longitudinal cohort study of anti-CCP positive RA patients, measuring levels of proinflammatory cytokines (IL-6 and TNF*α*), may be necessary to determine if these markers are predictive of a worse clinical outcome and comorbidities such as CVD.

## 5. Conclusion

We provide evidence that levels of anti-CCP and CRP are not only markers of poor clinical prognosis in RA but also evidence of increased cIMT that correlate with the AIP in these patients. Additional research with controlled cohorts is needed to confirm these results.

## Figures and Tables

**Table 1:** Comparison of ultrasound parameters between patients with rheumatoid arthritis (RA) and healthy controls (HC).

Ultrasound parameters	HC *n* = 62	RA *n* = 82	*P*
Common carotid artery			
Proximal third, mm ± SD	0.51 ± 0.11	0.59 ± 0.16	0.001
Distal third, mm ± SD	0.50 ± 0.13	0.66 ± 0.24	<0.001
Bulb, mm ± SD	0.58 ± 0.20	0.68 ± 0.23	0.01
Internal carotid artery			
Proximal third, mm ± SD	0.46 ± 0.12	0.60 ± 0.15	<0.001
Distal third, mm ± SD	0.43 ± 0.12	0.57 ± 0.17	<0.001
Increased carotid intima-media thickness, *n* (%)	9 (14.5)	35 (42.7)	0.005
Number of segments thickened, *n* (%)	1.53 ± 1.91	3.20 ± 2.16	<0.001
Presence of carotid plaque, *n* (%)	4 (6.5)	6 (7.3)	0.83

RA, rheumatoid arthritis; HC, healthy controls. Qualitative variables are expressed as frequencies (%); quantitative variables are expressed as means ± standard deviations (SD). Comparisons between proportions were computed using Chi-square or Fisher exact test. Comparisons between means were computed with unpaired Student's *t*-test.

**Table 2:** Characteristics and comparison of RA subgroups according to anti-CCP antibodies.

Variable	RA patients		*P*
	Anti-CCP (−) *n* = 37	Anti-CCP (+) *n* = 45	
Age, years ± SD	41.59 ± 11.41	44.09 ± 12.73	0.36
Disease duration, years ± SD	5.44 ± 7.69	4.90 ± 6.85	0.75
DAS28, units ± SD	1.43 ± 0.95	3.14 ± 0.44	0.05
Remission (<2.6)	19 (51.35)	16 (33.3)	0.06
Hands' Steinbrocker stage, III or IV, *n* (%)	0	7 (15.6)	0.01
*Lipid profile *			
TC, mg/dL	175.42 ± 39.74	203.02 ± 54.53	0.02
Tg, mg/dL	143.19 ± 61.70	165.51 ± 63.14	0.05
HDL-c, mg/dL	50.60 ± 16.68	42.98 ± 11.01	0.02
LDL-c, mg/dL	106.57 ± 30.28	103.49 ± 24.25	0.63
VLDL-c, mg/dL	28.72 ± 11.64	27.44 ± 12.97	0.66
AIP: TC/HDL-c	3.78 ± 1.34	5.11 ± 2.10	0.002
Low risk, *n* (%)	29 (78.4)	20 (44.5)	
Moderate risk, *n* (%)	7 (18.9)	15 (33.3)	0.006
High risk, *n* (%)	1 (2.7)	10 (22.2)	
*Serologic profile *			
ESR, mm/h	21.74 ± 3.16	27.09 ± 4.96	0.07
RF, IU/mL	111.86 ± 331.47	136.74 ± 201.57	0.73
CRP, mg/L	3.75 ± 2.00	11.47 ± 7.92	<0.001
TNF*α*, pg/mL	40.93 ± 3.14	66.78 ± 11.98	0.003
IL-6, pg/mL	20.92 ± 12.49	82.73 ± 29.87	<0.001
*DMARDs *			
Methotrexate, *n* (%)	31 (83.8)	45 (100)	0.04
Chloroquine, *n* (%)	22 (59.46)	32 (71.1)	0.22
Sulfasalazine, *n* (%)	9 (24.3)	9 (20.0)	0.79
Azathioprine, *n* (%)	6 (16.2)	8 (17.8)	1.00
Corticosteroids, *n* (%)	3 (8.1)	2 (4.4)	0.65

Anti-CCP, anticyclic citrullinated peptide antibodies; RA, rheumatoid arthritis; DAS28, disease activity score; TC, total cholesterol; Tg, triglycerides; HDL-c, high density lipoprotein cholesterol; LDL-c, low density lipoprotein cholesterol; VLDL-c, very low density lipoprotein cholesterol; AIP, atherogenic index of plasma; ESR, erythrocyte sedimentation rate; RF, rheumatoid factor; CRP, C-reactive protein; TNF*α*, tumor necrosis factor alpha; IL-6, interleukin-6; DMARDs, disease-modifying antirheumatic drugs.

Qualitative variables are expressed as frequencies (%); quantitative variables are expressed as means ± standard deviations (SD). Comparisons between proportions were computed using Chi-square or Fisher exact test. Comparisons between means were computed with unpaired Student's *t*-test.

**Table 3:** Correlation coefficients between cIMT and characteristics of evaluated the groups.

Baseline variable	cIMT (mm)	
	r	P

Age, years	0.587	<0.001
Disease duration, years	0.018	0.88
DAS28, units	0.350	0.05
TC, mg/dL	0.720	0.002
Tg, mg/dL	0.397	0.001
HDL-c, mg/dL	−0.595	0.02
LDL-c, mg/dL	0.332	0.007
VLDL-c, mg/dL	0.267	0.03
AIP: TC/HDL-c	0.716	0.001
ESR, mm/h	−0.137	0.38
RF, IU/mL	0.214	0.04
CRP, mg/L	0.799	<0.001
TNF*α*, pg/mL	0.642	0.001
IL-6, pg/mL	0.751	<0.001
Anti-CCP, U/mL	0.513	0.001

cIMT, carotid intima-media thickness; RA, rheumatoid arthritis; DAS28, disease activity score; TC, total cholesterol; Tg, triglycerides; HDL-c, high density lipoprotein cholesterol; LDL-c, low density lipoprotein cholesterol; VLDL-c, very low density lipoprotein cholesterol; AIP, atherogenic index of plasma; ESR, erythrocyte sedimentation rate; RF, rheumatoid factor; CRP, C-reactive protein; TNF*α*, tumor necrosis factor alpha; IL-6, interleukin-6; anti-CCP, anticyclic citrullinated peptide antibodies.

**Table 4:** Multiple linear regression analysis of cIMT with selected clinical features.

Independent variables	cIMT					
	Model 1		Model 2		Model 3	
	*β*	*P*	*β*	*P*	*β*	*P*
Age, years	0.002	0.09	0.005	0.12	0.001	0.71
Disease duration, years	−0.002	0.44	−0.008	0.24	−0.004	0.29
CRP, mg/L	0.006	0.05	0.008	0.05	0.008	<0.001
Anti-CCP, U/mL	0.001	0.005	0.001	0.009	0.001	0.03
RF, IU/mL	—	—	0.005	0.93	0.003	0.43
DAS28, units	—	—	−0.019	0.20	−0.046	0.08
TNF*α*, pg/mL	—	—	—	—	0.001	0.67
IL-6, pg/mL	—	—	—	—	0.003	0.05
*R* ^2^	0.87	<0.001	0.86	<0.001	0.89	<0.001

cIMT, carotid intima-media thickness; CRP, C-reactive protein; anti-CCP, anticyclic citrullinated peptide antibodies; RF, rheumatoid factor; DAS28, disease activity score; TNF*α*, tumor necrosis factor *α*, IL-6, interleukin-6; *R* ^2^, multiple coefficient of determination; *β*, standard regression coefficient.

**Table 5:** Published studies relating CVD risk factors and cIMT in RA.

Reference (author, year)	Study groups and design	CVD risk factors present in studied patients	Anti-CCP/IL-6/TNF*α*	Conclusions
del Rincón et al. (2003) [16]	Cross-sectional RA (*n* = 204) HC (*n* = 102)	Age ≥40 yrs old	No	ESR, CRP, and RF being associated with the increased cIMT in RA patients

Gerli et al. (2005) [22]	Cross-sectional RA (*n* = 101) HC (*n* = 75)	Systemic hypertension, dyslipidemia, type 2 DM, current smokers, and family history of CVD	No	Smoking increasing the cIMT in RA patients

Hannawi et al. (2007) [23]	Cross-sectional RA (*n* = 40) HC (*n* = 40)	Current smokers	No	Higher cIMT in RA than HC; cIMT correlating with DAS28 and CRP; atherosclerotic plaques being more frequent in RA

Ciftci et al. (2008) [24]	Cross-sectional RA (*n* = 30) HC (*n* = 52)	None	No	Increased cIMT in RA versus HC; correlation between cIMT and time of evolution

Stamatelopoulos et al. (2009) [25]	Cross-sectional RA (*n* = 84) HC (*n* = 84) Type 2 DM (*n* = 48)	None	No	Increased cIMT in RA versus HC

Ristić et al. (2010) [26]	Cross-sectional RA (*n* = 42) HC (*n* = 32)	Current smokers	Anti-CCP	Higher cIMT in RA smokers; negative correlation with time on treatment; positive correlation with FR and ESR

Ahmed et al. (2010) [27]	Cross-sectional Early RA (*n* = 40) HC (*n* = 40)	None	No	DAS28, ESR, CRP, disease duration, steroids use, and ox-LDL associated with the presence of plaque

Södergren et al. (2010) [4]	Cross-sectional RA (*n* = 79) HC (*n* = 44)	Current smokers, family history of CVD	No	Higher cIMT in RA versus HC

El-Barbary et al. (2011) [28]	Cross-sectional RA (*n* = 100) HC (*n* = 100)	None	IL-6 TNF*α* Anti-CCP Anti-MCV	Positive correlation between anti-MCV with changes in cIMT, not with anti-CCP

Ajeganova et al. (2012) [29]	Prospective cohort RA	None	No	Higher cIMT associated with CRP

Targońska-Stepniak et al. (2011) [30]	Cross-sectional RA (*n* = 74) HC (*n* = 31)	Type 2 DM, Systemic hypertension	Anti-CCP	Higher cIMT associated with age, anti-CCP, erosions, and extra-articular manifestations

Akrout et al. (2012) [31]	Cross-sectional RA (*n* = 34) versus HC (*n* = 34)	None	No	Higher cIMT in RA than control; higher AIP in RA and lower HDL

Vázquez-Del Mercado Mónica (Present study)	Cross-sectional RA (*n* = 60) versus HC (*n* = 62)	None	Anti-CCP IL-6 TNF*α*	Levels of anti-CCP and CRP associated with the cIMT in RA in multiple linear regression

CVD, cardiovascular disease; DM, diabetes mellitus; RA, rheumatoid arthritis; HC, healthy controls; cIMT, carotid intima-media thickness; ESR, erythrocyte sedimentation rate; RF, rheumatoid factor; CRP, C-reactive protein; TNF*α*, tumor necrosis factor *α*; IL-6, interleukin-6; anti-CCP, anticyclic citrullinated peptide antibodies; ox-LDL, oxidized low density lipoprotein; anti-MCV, antimutated citrullinated vimentin; DAS28, disease activity score.

## Abbreviations

Anti-CCP: Anticyclic citrullinated peptides
cIMT: Carotid intima-media thickness
AIP: Atherogenic index of plasma
TNF*α*: Tumor necrosis factor alpha
IL-6: Interleukin-6
HC: Healthy control
DAS28: Disease activity score 28
HDL-c: High density lipoprotein cholesterol
CRP: C-reactive protein
RA: Rheumatoid arthritis
CVD: Cardiovascular disease
RF: Rheumatoid factor
ESR: Erythrocyte sedimentation rate
CCA: Common carotid artery
MCP: Metacarpophalangeal joints
US: Ultrasound
ICA: Internal carotid artery
BF: Carotid bifurcation
TC: Total cholesterol
Tg: Triglycerides
LDL-c: Low density lipoprotein cholesterol
SD: Standard deviations
OR: Odds ratio
Anti-MCV: Antimutated citrullinated vimentin
DMARDs: Disease-modifying antirheumatic drugs.
